# Thermally Solvent-Free Cross-Linked pH/Thermosensitive Hydrogels as Smart Drug Delivery Systems

**DOI:** 10.3390/gels10120834

**Published:** 2024-12-18

**Authors:** Sanda Bucatariu, Bogdan Cosman, Marieta Constantin, Gabriela Liliana Ailiesei, Daniela Rusu, Gheorghe Fundueanu

**Affiliations:** 1 “Petru Poni” Institute of Macromolecular Chemistry, Grigore Ghica Voda Alley 41A, 700487 Iasi, Romania; marieta@icmpp.ro (M.C.); gdarvaru@icmpp.ro (G.L.A.); rusu.daniela@icmpp.ro (D.R.); 2 Stem Cell Biology Laboratory, Institute of Cellular Biology and Pathology “Nicolae Simionescu” of the Romanian Academy, 8 B. P. Hasdeu Street, District 5, 050568 Bucharest, Romania; cosman.bogdan@icmpp.ro

**Keywords:** drug delivery systems, cross-linked polymers, pH/thermosensitive hydrogels

## Abstract

An imbalance in the body’s pH or temperature may modify the immune response and result in ailments such as autoimmune disorders, infectious diseases, cancer, or diabetes. Dual pH- and thermo-responsive carriers are being evaluated as advanced drug delivery microdevices designed to release pharmaceuticals in response to external or internal stimuli. A novel drug delivery system formulated as hydrogel was developed by combining a pH-sensitive polymer (the “biosensor”) with a thermosensitive polymer (the delivery component). Thus, the hydrogel was created by cross-linking, using a solvent-free thermal approach, of poly(N-isopropylacrylamide-co-N-hydroyethyl acrylamide), P(NIPAAm-co-HEAAm), and poly(methylvinylether-alt-maleic acid), P(MVE/MA). The chemical structure of the polymers and hydrogels was analyzed using Fourier-transform infrared (FTIR) and proton nuclear magnetic resonance (^1^H NMR) spectroscopies. The pH/thermosensitive hydrogel loses its thermosensitivity under physiological conditions but, remarkably, can recover the thermosensitive capabilities when certain physiologically active biomolecules, acting as triggering agents, electrostatically interact with pH-sensitive units. Our research aimed to develop a drug delivery system that could identify the disturbance of normal physiological parameters and instantaneously send a signal to thermosensitive units, which would collapse and modulate the release profiles of the drug.

## 1. Introduction

Drug delivery systems (DDS) improve the effectiveness of treatment by enhancing the characteristics of the current drug, such as its solubility, biocompatibility, and bioavailability. Typically, there are three primary categories of drug delivery systems, which are mainly composed of polymeric or copolymeric materials: tablet, by physically combining the drug with a polymer matrix; capsule, by entrapping the drug within a polymer shell; and bioconjugate, by chemically linking the drug to a polymer or copolymer [1,2].

Hydrogels are versatile biomaterials and have demonstrated significant potential in various sectors, such as drug delivery [1,3,4,5], tissue engineering [6], implants [7,8], biosensing [9], and regenerative medicine [1,10]. Among these, thermosensitive hydrogels occupy a special place because they mimic the behavior of living organisms by changing their inherent properties at small changes in the temperature of the human body.

Several stages are involved in the synthesis of thermosensitive hydrogels. Acrylamide monomers such as N-isopropylacrylamide (NIPAAm) are utilized to produce hydrogels that exhibit temperature sensitivity [4,5]. The copolymerization of NIPAAm with pH-sensitive monomers (usually weak acids or weak bases) results in the formation of copolymers sensitive to both pH and temperature [11,12]. For instance, poly(N-isopropylacrylamide) (PNIPAAm) serves as a temperature-responsive segment, whereas polymeric blocks with chemical functions such as amines, acids, acetals, amino alkylmethacrylates, ortho esters, vinyl esters, and hydrazones can operate as pH-responsive segments [11,12]. Temperature- and pH-responsive polymers exhibit physicochemical alterations in response to variations in the pH and temperature of the surrounding medium [11].

Usually, the monomers are polymerized through free radical polymerization utilizing initiators and accelerators such as ammonium persulfate and tetramethylethylenediamine (TEMED), respectively. To form a three-dimensional network, cross-linking agents like N,N’-methylenebisacrylamide (BisAAm) are utilized [1]. Chemical cross-linking is a flexible and adaptable technique for adjusting polymer microstructure and physical properties with excellent mechanical stability [13]. However, most chemical cross-linking agents are toxic compounds for the body; as a result, there is a growing emphasis on environmentally friendly approaches in the creation of hydrogels to prevent the use of hazardous chemicals [14].

Poly(methyl vinyl ether-alt-maleic anhydride) (P(MVE/MAn)) copolymer possesses properties characteristic of maleic anhydride (MAn) copolymers, being a safe biomaterial for humans and animals, exhibiting favorable hydrophilicity, chemical stability, and biocompatibility [5]. Additionally, the hydrolyzed form, P(MVE/MA) copolymers, offers two significant advantages that make them suitable for intestinal drug delivery: mucoadhesiveness and pH-sensitiveness. The maleic acid copolymers with strong bioadhesive properties have a significantly low level of oral toxicity [5].

In a range of biomedical applications, the adhesive properties of hydrogels are of particular interest. Initially, it is important to note that the interface between the hydrogel and tissue typically exhibits a moist condition [15]. The presence of water molecules at the interface creates a hydration layer that affects the molecular-level forces. Additionally, the macroscopic interfacial water decreases the effective area of the adhesion surface [16]. Furthermore, the tissue surface exhibits elasticity and a delicate texture, allowing for a wide range of movements. Adhesion must possess both high strength and stretchability while avoiding hard interfaces or detachment during movement. Furthermore, it is imperative for bioadhesive hydrogels to exhibit excellent biocompatibility, preventing them from inducing chemical toxicity or mechanical harm to the tissue [5]. The supple and moist characteristics of hydrogels provide challenges in generating accurate micro-nano structures that closely resemble the sticky organs seen in natural animals. Nevertheless, the distinctive characteristics of hydrogels, such as their capacity to expand and respond to changes in temperature, provide artificial adhesion materials with greater flexibility in design and enhanced functional capabilities. This enables them to potentially outperform natural materials in terms of controllability and adhesive properties [16].

The freeze-drying technique, also known as lyophilization, has been widely used to create porous scaffolds. This method utilizes fast cooling to identify thermodynamic instability in the system and induce phase separation. Subsequently, water is extracted through sublimation in a vacuum, resulting in the formation of pores in the areas it previously inhabited. Freeze-dried scaffolds are becoming more commonly utilized for the creation of 3D-linked micropores, which play a significant role in the transportation of biomolecules and cells. Analysis of the SEM micrographs revealed the presence of highly porous and well-connected three-dimensional structures [17].

In this work, a novel solvent-free cross-linking technique was used to create the pH/temperature-sensitive hydrogel based on thermosensitive polymer P(NIPAAm-co-HEAAm) and pH-sensitive polymer P(MVE/MA) in the proper ratio. Under physiological conditions at pH = 7.4, the carboxylic groups of P(MVE/MA) are ionized and much more hydrophilic, and therefore the hydrogel loses the thermosensitive properties. However, after electrostatic interactions of carboxylic groups, with some model biomolecules playing the role of a triggering agent (diphenhydramine, DPH), the delivery component (thermosensitive polymer) restores the thermosensitive properties and undergoes collapse, slowing drug diffusion.

## 2. Results and Discussion

### 2.1. Synthesis and Characterization of P(NIPAAm-co-HEAAm) (Shortly H in Sample Codes)

**Figure 1:**
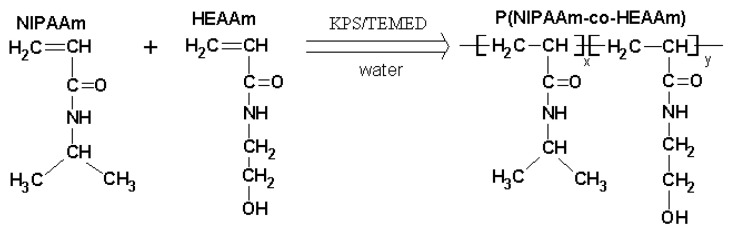
Chemical reactions involved in the synthesis of P(NIPAAm-co-HEAAm).

It is well-known that NIPAAm possesses a sharp phase transition (lower critical solution temperature, LCST) at a temperature of around 32 °C [12], which is slightly below the temperature of the human body. In order to increase the LCST toward the body temperature, NIPAAm is usually copolymerized with hydrophilic monomers [18,19]. Here, hydroxyethyl acrylamide (HEAAm) in various molar ratios was first selected to be copolymerized with NIPAAm by free radical polymerization in water (Table 1). The redox system, including potassium persulfate (KPS) and N,N,N′,N′-tetramethylethylenediamine (TEMED), was employed to initiate the polymerization reaction (Figure 1).

Secondly, the HEAAm molecule was selected as a co-monomer due to its secondary alcohol group, which can be utilized for covalent cross-linking purposes. Thirdly, the chemical structure of the HEAAm (Figure 1) guarantees the presence of a continuous acrylamide sequence similar to that of NIPAAm, which in turn preserves the thermosensitive characteristics of the copolymers [18].

**Figure 2:**
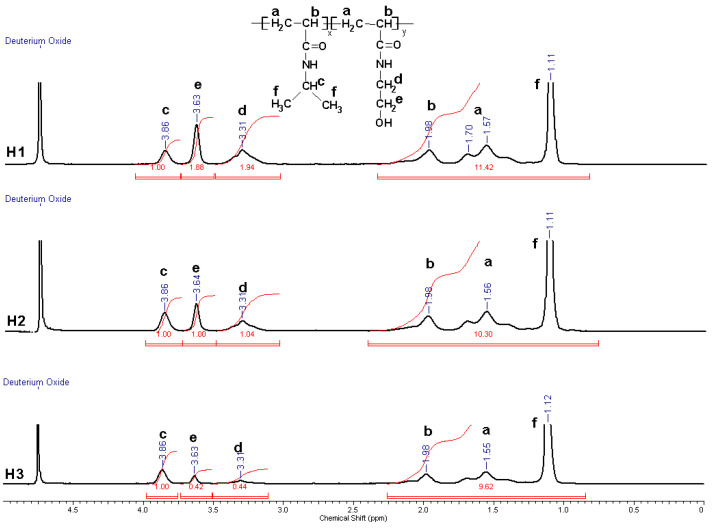
^1^H NMR spectrum of P(NIPAAm-co-HEAAm) (samples H1, H2, and H3 in Table 1).

The ^1^H-NMR spectra, as shown in Figure 2, confirms the formation of the copolymers. Also, ^1^H-NMR analysis was used to determine the composition of the P(NIPAAm-co-HEAAm) copolymer. Using Equation (1), the molar fraction of HEAAm in P(NIPAAm-co-HEAAm) was determined [20], as follows:

(1) $$\%\;HEAAm=\frac{\frac{A_{3.31ppm}}{2}}{\frac{A_{3.86ppm}}{1}+\frac{A_{3.31ppm}}{2}}\times 100,$$

where $\frac{A_{3.86ppm}}{1}$ and $\frac{A_{3.31ppm}}{2}$ represent the molar fraction of NIPAAm and HEAAm determined by calculating the area of the methynic and methylenic protons at 3.86 and 3.31 ppm, respectively.

The proportion of co-monomers in the copolymer closely corresponds to the proportions of co-monomers in the initial feed, as indicated in Table 1. The variation of molar ratio between the co-monomers led to the copolymers with different LCST values, as depicted in Table 1. The relationship between the LCST and the amount of HEAAm is directly proportional, meaning that the higher the proportion of HEAAm in the copolymer. the greater the LCST measured in the PBS.

#### Phase Transition Characterization

**Figure 3:**
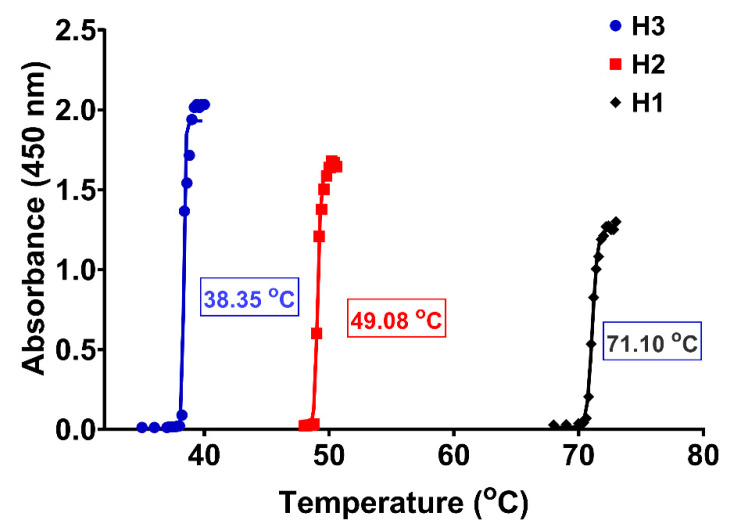
LCST profiles of P(NIPAAm-co-HEAAm) in PBS at different NIPAAm/HEAAm molar ratios (see Table 1). The concentration of copolymers was 1% (*w*/*v*).

Displaying a phase transition around the human body temperature in a narrow temperature range is a significant characteristic of a thermosensitive polymer planned for biomedical applications [4]. This study uses the cloud point method to determine the LCST, which is the temperature when the first opalescence (absorbance value of 0.5) occurs in the polymer aqueous solution. The H3 copolymer appears to be suitable for biomedical applications because, under physiological conditions at pH = 7.4, it possesses a phase transition at 38.3 °C. Furthermore, the transition is extremely abrupt, taking place within a range of no more than two Celsius degrees (Figure 3).

### 2.2. Synthesis and Characterization of Hydrogels

#### 2.2.1. Influence of P(MVE/MA) on the LCST of P(NIPAAm-co-HEAAm)

**Figure 4:**
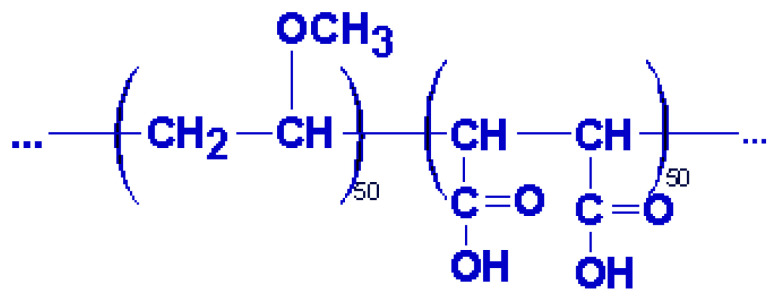
Chemical structures of P(MVE/MA).

Poly((methyl vinyl ether)-alt-(maleic acid)) P(MVE/MA) represents an attractive polymer since it possesses free carboxylic groups (Figure 4), which can form amide and ester linkage. On the other hand, this polymer has many important applications in biotechnology, pharmacology, and health care due to its hydrophilicity, biocompatibility, and bioadhesiveness [21].

**Figure 5:**
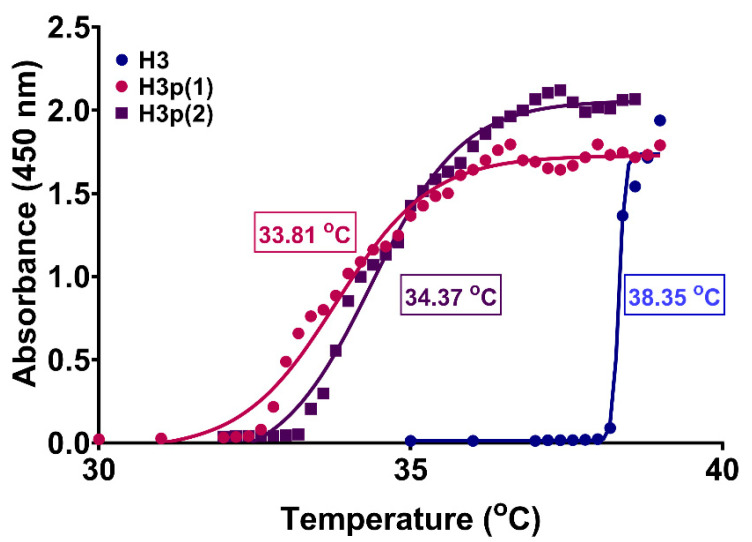
LCST profiles of P(NIPAAm-co-HEAAm)/P(MVE/MA) mixture in PBS at different H/p ratios (*w*/*w*) (see Table 2). For comparison, the LCST profile of P(NIPAAm-co-HEAAm) is given. The concentration of the polymer mixture was 1% (*w*/*v*).

It must be underlined that the solutions obtained by mixing the H3 and P(MVE/MA) with low molecular weight, Mw = 210,000 g/mol (coded “p”), became opaque at a temperature lower than that of H3 alone. Thus, we studied the influence of the amount of p on the LCST of the mixture solution. From Figure 5, it can be observed that the polymer p decreased the value of LCST and, at the same time, increased the transition temperature range.

**Figure 6:**
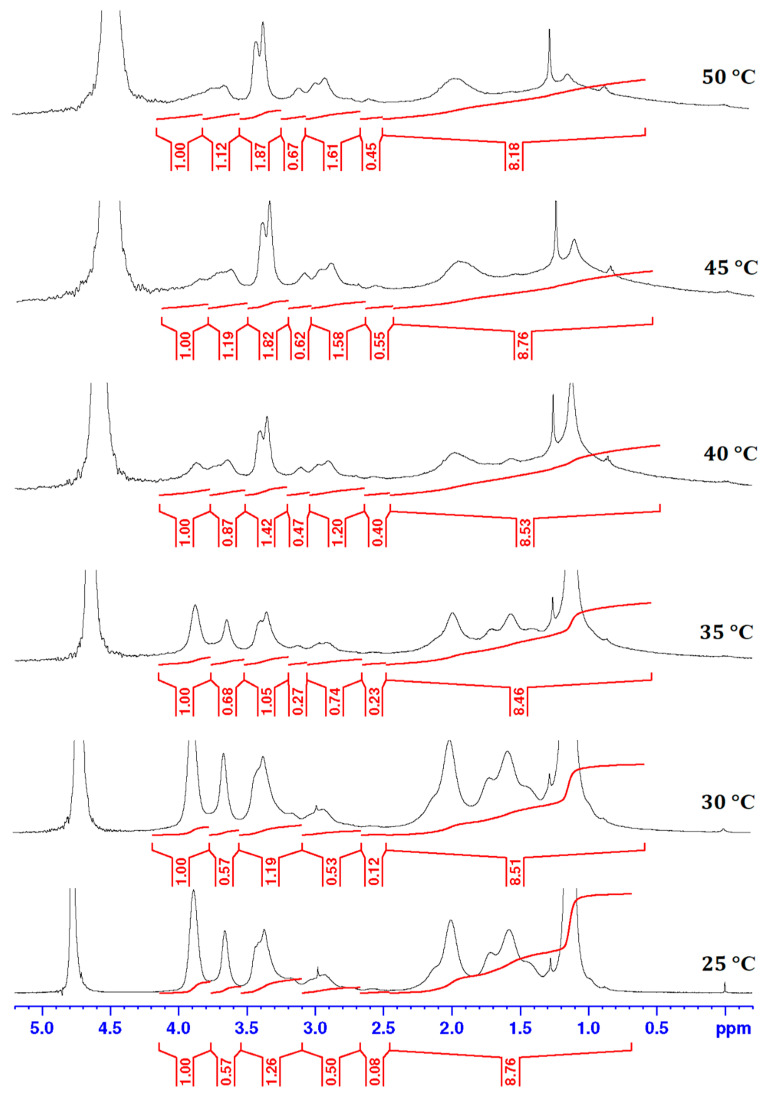
^1^H-NMR spectra of P(NIPAAm-co-HEAAm) and P(MVE/MA) mixture (2:1, *w*/*w* weight ratio) in D_2_O (see Table 2) at different temperatures.

In order to understand the influence of P(MVE/MA) on conformation of the P(NIPAAm-co-HEAAm) macromolecular chains below and above the LCST, ^1^H-NMR spectra at different temperatures were recorded. Therefore, below the LCST at 25 °C, for the mixture of 2:1 weight ratio between P(NIPAAm-co-HEAAm) and P(MVE/MA) (sample H3p(2)), the spectrum is similar to that of the thermosensitive polymer alone, the signals of *p* being covered (Figure 6). On the opposite, as the temperature increases, the NIPAAm peaks decrease and the P(MVE/MA) peaks appear. As follows, a progressive reduction of the methine peak strength of NIPAAm is observed as the temperature rises until its almost complete disappearance (Figure 6). This effect results from a reduction in the molecular mobility of the isopropyl segments as the temperature increases. The peaks begin to diminish around 35 °C and are poorly perceptible by 50 °C. The relative intensity of the methine peak to the methylenic signal increases with elevated temperature, indicating that the polar -CH_2_OH groups of HEAAm cover up the isopropyl groups. This could explain why the LCST values decrease in the mixture solution.

#### 2.2.2. Hydrogel Synthesis and Characterization

For preparing pH/thermosensitive hydrogels (Hp and HP), H3 thermosensitive sample and pH-sensitive P(MVE/MA) (resulted from the hydrolysis of MAn) with two different molar masses were used, a low one of 210 kDa (p) and a high one of 1000 kDa (P) (Table 2). The two copolymers were mixed together in water to achieve a final solution of polymers with a concentration of 3% (*w*/*v*). Considering the molar ratios between the functional groups of the two copolymers, only two H:P(p) weight ratios 1:1 and 2:1, simplified 1 and 2, respectively, which are specified in parentheses, were considered.

**Table 2:** Feed compositions and main characteristics of the most relevant P(NIPAAm-co-HEAAm)/P(MVE/MA) cross-linked hydrogels.

**Sample** **Code**	**Initial Feed Composition** **(%, *w*/*v*)**			**Physicochemical Characteristics**		
	**H3**	**p**	**P**	**E.C.** * **(meq/g)**	**C.D.** ** **(%)**	**Swelling Degree** **(g/g)**
H3p(1)	1.5	1.5	-	4.92 ± 0.31	14.30 ± 0.42	66.69 ± 2.31
H3p(2)	2	1	-	2.91 ± 0.11	23.82 ± 0.81	56.65 ± 1.84
H3P(1)	1.5	-	1.5	2.13 ± 0.10	44.40 ± 1.11	30.62 ± 0.93
H3P(2)	2	-	1	3.16 ± 0.22	17.46 ± 0.56	33.16 ± 1.21

Data are the results of three independent experiments ± SD. * E.C. is the exchange capacity = milliequivalents of -COOH/gram of hydrogels. ** The cross-linking degree (C.D.) is defined as the ratio of the difference between the theoretical and actual exchange capacity divided by theoretical exchange capacity.

**Figure 7:**
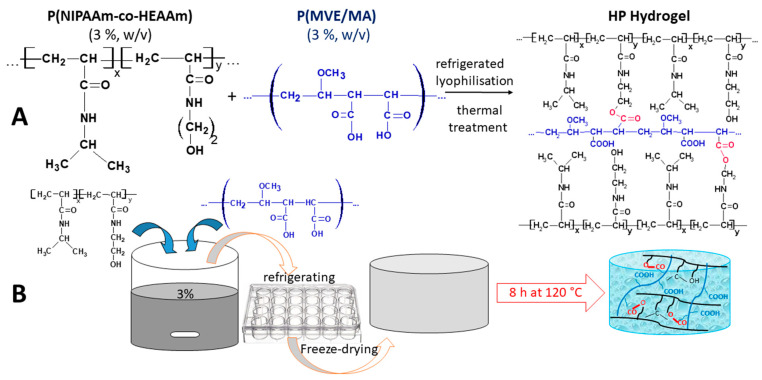
(**A**) Proposed cross-linking mechanism between P(NIPAAm-co-HEAAm) and P(MVE/MA); (**B**) Schematic representation of Hp hydrogel synthesis.

Aqueous solutions of P(NIPAAm-co-HEAAm) and P(MVE/MAn) (1:1 and 2:1, respectively, weight ratio) were combined in a preliminary experiment. The mixed solution was poured into 24-well plates, refrigerated, and subjected to freeze-drying for a duration of 2 days (Figure 7).

It is important to mention that, in an initial trial, the polymer mixture was rapidly frozen using liquid nitrogen. However, after the process of lyophilization, the resulting samples, in cylindrical shape, were found to be fragile and easily breakable. Therefore, the practice of gradually freezing in the freezer was determined to be the most successful method for producing soft and relatively stable samples. However, physically cross-linked samples still exhibited instability in water as a result of the disintegration of the polymer chains. Consequently, heat treatment was used to form interchain ester cross-links, rendering the lyophilized polymer blends insoluble. To achieve cross-linked hydrogels, the samples were subsequently subjected to one or two rounds of 8 h heat treatment at 120 °C inside the oven. At this temperature, both the esterification and anhydrization took place. The esterification involves the reaction between the hydroxyl group from P(NIPAAm-co-HEAAm) and the carboxylic groups of P(MVE/MA), while the anhydrization supposes the reaction between two carboxylic groups in the molecule of P(MVE/MA). The hydrogels obtained were thoroughly rinsed with a substantial volume of water to eliminate the soluble portion of polymer fragments. Hydrolysis of the anhydride bonds occurs during the washing process, leaving behind only the ester bonds that contribute to the stability of the hydrogel network.

**Figure 8:**
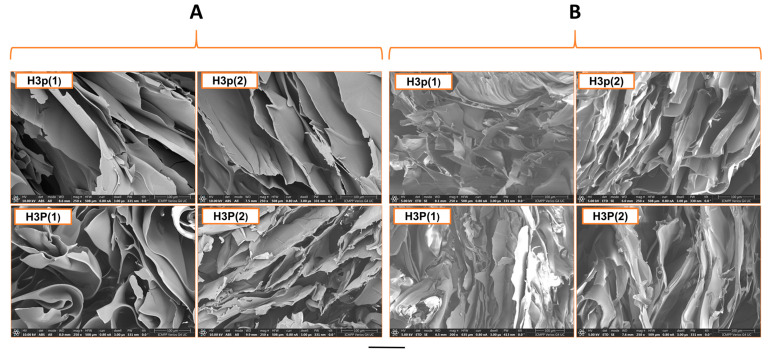
Scanning electron micrographs of pH/thermosensitive hydrogels thermally treated (8 h at 120 °C) for one round (**A**) and two rounds (**B**). Bar corresponds to 100 μm.

The SEM pictures demonstrate a clear cross-sectional morphology of the lyophilized samples, displaying a multilayered appearance and porous structures (Figure 8). It is noteworthy that, in contrast to hydrogels composed of poly(NIPAAm-co-HEAAm) cross-linked with BisAAm, which exhibit a homogeneous porous structure as reported in a prior study [22], the hydrogels cross-linked with P(MVE/MA) possess a lamellar structure characterized by pores formed between the layers. The samples obtained with a double amount of thermosensitive polymer (Hp(2) and HP(2)) exhibit more pores between layers. Also, it can be observed that the hydrogel obtained with a high molar mass of P(MVE/MA) (HP) had much smaller pores with irregular structures and a denser network than hydrogel obtained with the copolymer with low molar mass. Furthermore, the hydrogels that underwent two rounds of thermal treatment (Figure 8B), intended to enhance ester bond formation, appear to deteriorate rather than show increased cross-linking. The existence of tiny pores within larger cavities demonstrates pore connectivity, a beneficial characteristic for the capillary movement of biological fluids and drugs across the polymer matrix [5].

**Figure 9:**
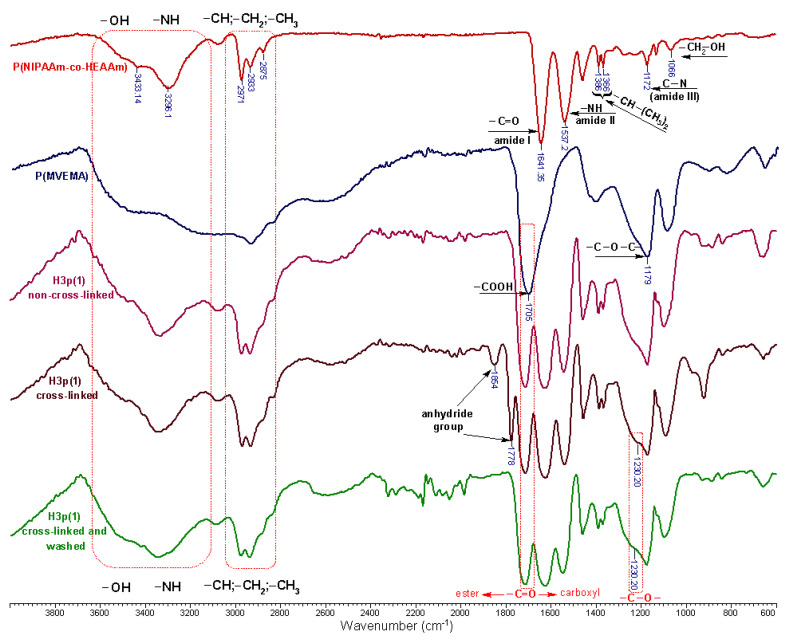
FTIR spectra of P(NIPAAm-co-HEAAm), P(MVE/MA), and H3p(1) samples (non-cross-linked; cross-linked; cross-linked and washed).

Fourier Transform Infrared Spectroscopy (FTIR) is an effective instrument for examining precise interactions between polymers [13]. An analysis of the interfacial contact was conducted using ATR-FTIR for all compositions. These analyses were performed to understand the extent of inter-polymer complexation that can occur as a result of hydrogen bonding between the carbonyl (C=O) groups of P(MVE-MA) and the hydroxyl (OH) groups of P(NIPAAm-co-HEAAm). Figure 9 displays the FTIR spectra of P(NIPAAm-co-HEAAm), P(MVE/MA), and P(NIPAAm-co-HEAAm)/P(MVE-MA) blends both before and after thermal cross-linking.

The FT-IR spectrum of P(NIPAAm-co-HEAAm) shows distinct absorption bands at specific frequencies. These include a band at 1641 cm^−1^, which corresponds to the stretching of the C=O bond in the amide I. Another band at 1537 cm^−1^ corresponds to the bending of the N–H bond in amide II and a band at approximately 1172 cm^−1^, which is associated with the C–N bond in amide III. Moreover, there are bands at around 1366/1386 cm^−1^ that are attributed to the isopropyl groups (CH–(CH_3_)_2_). The absorption bands observed at 1458 cm^−1^ are ascribed to the bending of C–H bonds in –CH(CH_3_)_2_ and –CH_2_– groups. The peak observed at 1066 cm^−1^ in the spectra is attributed to the C–O stretching of primary alcohol. The absorption band spanning from 2870 to 3000 cm^−1^ is indicative of C–H stretching vibrations associated with –CH_3_ and –CH_2_– groups. The broad spectral bands observed between 3100 and 3600 cm^−1^, with distinct peaks at 3296 cm^−1^ and 3433 cm^−1^, can be attributed to the valence vibrations of the N–H and O–H functional groups [3,23].

The P(MVE/MA) spectrum has distinct bands around 1705 and 1179 cm^−1^, which can be attributed to the presence of the COOH and C–O–C groups, respectively [13]. Moreover, the bands in the range of 2940 to 2845 cm^−1^ were identified as C–H stretching vibrations of CH, CH_2_, and CH_3_ groups in the P(MVE/MA). They were then followed by a broadband signal peculiar to OH groups at 3100 and 3436 cm^−1^ [5]. The spectra of all copolymers and mixtures exhibit a band in the range of 1650–1800 cm^−1^, which originates from the C=O functional group [13]. The OH group bands of both P(NIPAAm-co-HEAAm) (3433 cm^−1^) and P(MVE/MA) (3436 cm^−1^) gradually expand and shift at around 3500 cm^−1^ in the thermally cross-linked copolymers. This change is the result of the robust interaction between hydroxyl groups on P(NIPAAm-co-HEAAm) and carboxyl groups on P(MVE/MA). The peak at 1705 cm^−1^ attributed to the carbonyl groups of acidic groups of P(MVE/MA) is shifted to a higher wavenumber (1715 cm^−1^) in all copolymer mixtures, cross-linked or not. In addition, the peaks at 1537 and 1641 cm^−1^ attributed to carbonyl groups of the amide I and II are shifted at 1543 and 1627 cm^−1^, respectively. Moreover, the FTIR spectra also confirmed the anhydride bonds in the unwashed thermally cross-linked hydrogel samples by the appearance of new two bands at 1778 and 1854 cm^−1^. In the washed samples, these bands disappear due to the hydrolization of the anhydride groups. This observation suggests that a cross-linking reaction took place between P(NIPAAm-co-HEAAm) and P(MVE/MA), resulting in the formation of new covalent bonds. The bands corresponding to the COOH groups exhibit both a shift and an increase in intensity when P(MVE/MA) is initially combined with P(NIPAAm-co-HEAAm). The thermal cross-linking leads to the emergence of a peak at approximately 1715 cm^−1^, which is indicative of the vibration of the COOH group. Additionally, the P(NIPAAm-co-HEAAm)/P(MVE/MA) hydrogels exhibit a novel absorption band at around 1230 cm^−1^ for C–O stretch mode of ester group in cross-linked samples. The C=O streching of ester bound should be at 1615 cm^−1^, but it overlaps with the carbonyl bands of the amide II (1641 cm^−1^) in P(NIPAAm-co-HEAAm), which is shifted toward lower wavenumbers due to the formation of ester bonds (CO–O) between the alcohol group of P(NIPAAm-co-HEAAm) and the carboxyl group of P(MVE-MA) [13]. Moreover, the formation of ester cross-linkages was also confirmed by comparing the FTIR spectra of a 0.1 M NaOH-treated sample with an untreated sample, as summarized in Figure 9. The peaks observed at 1712.7 cm^−1^ of the untreated sample could be due to both the ester linkage and the protonated carboxylic acid groups. In the NaOH-treated sample, the remaining signal at 1709 cm^−1^ solely confirmed the establishment of ester cross-linkages between P(NIPAAm-co-HEAAm) and P(MVE/MA) [24].

#### 2.2.3. Swelling/Deswelling Behavior

The primary characteristic of smart hydrogels that is widely recognized is the amplitude of the volume variation in response to environmental stimuli. As the hydrogels are designed for biomedical applications, swelling studies were carried out under pseudo-physiological conditions. The swelling properties of the hydrogel depend on the carboxylic group content (exchange capacity). Thus, due to the un-reacted carboxylic groups in the cross-linking stage, the H3p(1) and H3P(2) samples still possess a high exchange capacity (4.92 and 3.16 meq/g, respectively), as determined by conductometric titration; the theoretical exchange capacity (before cross-linking) being 5.75 and 3.83 meq/g for H3p(1) and H3P(2), respectively (see Table 2). Accordingly, the HP swelling capacity will be deeply affected by the ionization of the carboxylic groups [25], considering that P(MVE/MA) has two pKa values (pKa1 = 3.47; pKa2 = 6.47) [26]. Furthermore, the degree of cross-linking (C.D.) was indirectly determined by the conductometric titration of carboxylic groups. Thus, the C.D. was estimated based on the difference between the theoretical exchange capacity and the effective exchange capacity determined by conductometric titration, and the results are reported in Table 2.

**Figure 10:**
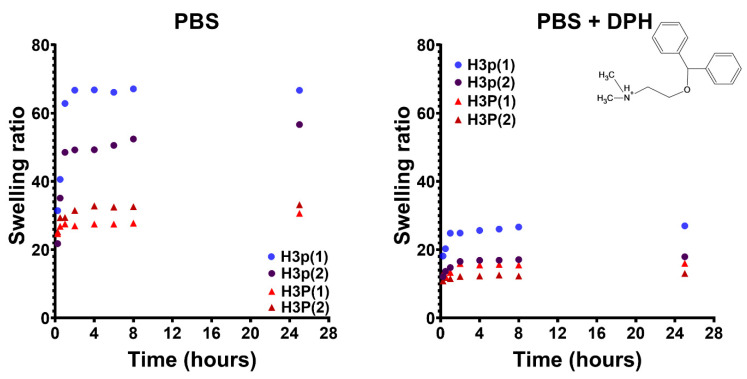
Swelling kinetics of P(NIPAAm-co-HEAAm)/P(MVE/MA) cross-linked hydrogels in phosphate buffer solution at pH 7.4 at 37 °C, without/with a stoichiometric amount of DPH.

Swelling kinetics is another important characteristic of pH/temperature-sensitive hydrogels for biomedical applications. In this study, the swelling kinetics were investigated under physiological conditions in the absence and presence of an equimolar amount of a model biomolecule (diphenhydramine, DPH) with the role of a triggering agent. It can be seen from Figure 10 that the swelling ratio of the hydrogels is lower in the presence than in the absence of DPH. This behavior can be explained by the formation of both electrostatic and hydrophobic interactions between the hydrogels and DPH, the last one acting as an additional cross-linker. Moreover, the hydrogel obtained using the higher mass P(MVE/MA) copolymer has a lower swelling ratio, probably due to the more pronounced interactions between the two copolymers and therefore to the denser and less flexible network.

**Figure 11:**
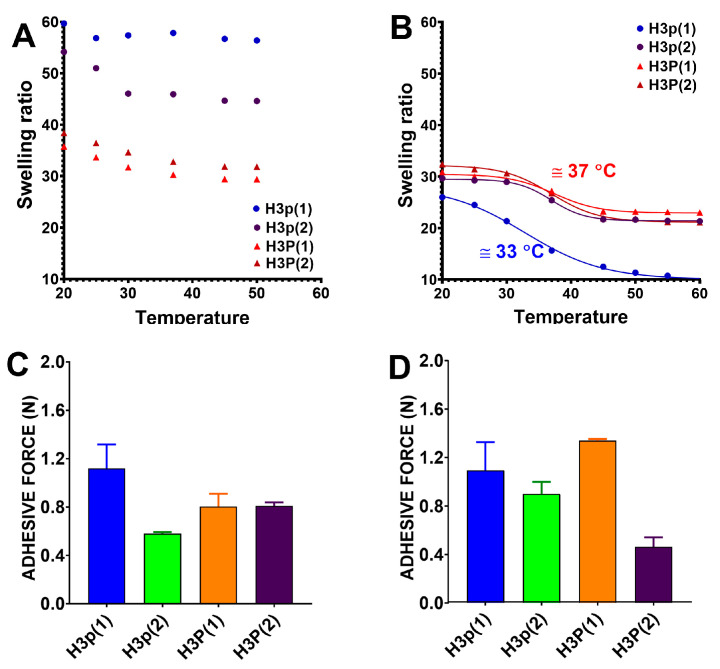
Swelling ratios of P(NIPAAm-co-HEAAm)/P(MVE/MA) hydrogels as a function of temperature in PBS (pH = 7.4) (**A**) and PBS + DPH (DPH:COOH = 1:1, molar ratio) (**B**). Bioadhesive properties of pH/thermosensitive hydrogels in PBS (**C**) and PBS + DPH (**D**).

In order to determine the VPTT of hydrogels, the variation of the swelling degree with temperature was studied in PBS at pH = 7.4 and in the absence or presence of DPH. In the absence of the triggering agent (DPH), the hydrogels obtained both with copolymer (P(MVE/MA)) with low and high mass do not show thermosensitivity, or it is very low (Figure 11A). At this pH, the carboxylic groups of P(MVE/MA) are ionized (pKa1 = 3.47; pKa2 = 6.47), and their strong hydrophilic contribution determines the loss of thermosensitive properties. On the opposite, the hydrogels exhibit thermosensitivity when there are electrostatic interactions between the carboxylic groups of P(MVE/MA) in the copolymer and the amine groups of DPH (Figure 11B). In fact, after electrostatic interactions with DPH, the complex of the drug-copolymer acts as a new copolymer with a hydrophilic/hydrophobic balance different than that of the un-complexed one. This phenomenon has also been documented in previous research employing hydrogels based on copolymers that are responsive to both pH and temperature [12,27].

The occurrence of thermosensitivity following the electrostatic interactions between carboxylic groups and positively charged hydrophobic molecules gives a sensor characteristic to the hydrogels. The carboxylic groups can interact with certain molecules, modifying the hydrophilic/hydrophobic balance of the hydrogel and imparting thermosensitivity. The pH-sensitive copolymer functions as a sensor, while the thermosensitive polymer serves as an actuator. This type of hydrogel is “activated” at normal body temperature upon engagement with the positively charged hydrophobic molecules, namely the “triggering agent”, in contrast to typical thermosensitive hydrogels that collapse when exposed to elevated temperatures [12].

From a biological perspective, the gradual shrinking of the hydrogel may influence the release of the drugs previously encapsulated within it.

#### 2.2.4. Bioadhesiveness

In order to assess the interaction between P(NIPAAm-co-HEAAm)/P(MVE/MA) hydrogels and the skin surface, bioadhesive tests were conducted and presented in Figure 11C,D. This study compared the mucoadhesive performance of different samples using maximum detachment force (Fmax). Various surface substrates, including porcine stomach tissue, chicken pouch tissue, bovine sublingual mucosa, bovine duodenal mucosa, mucin disc, and mucin gel, have been employed as model substrates in texture analysis [28]. In this experiment, a chicken skin mucosa was used as the surface model. It is generally accepted that the required properties of a polymer (hydrogel) to be chosen as bioadhesive material are the possession of (i) functional groups with strong hydrogen bonding capacity, such as hydroxyl and carboxyl, (ii) high molecular mass, (iii) great polymer hydration, and (iv) chain flexibility [29]. As follows, the hydrogel H3p(1) with the largest number of carboxyl groups (highest exchange capacity = 4.92 meq/g) and the highest swelling degree (66.69 g/g) displays the greatest adhesive force. On the opposite, the hydrogel H3p(2) with almost the same swelling degree (56.65 g/g) but with a lower number of carboxylic groups (exchange capacity = 2.91 meq/g) shows the smallest adhesive force. Hydrogels H3P(1) and H3P(2) with high molecular mass of P(MVE/MA) but with low swelling degrees (30.62 and 33.16 g/g, respectively) and intermediate values of exchange capacities display an intermediate value of adhesive forces. Controversially, excess polymer hydration led to a reduction in the strength of the polymer–mucosa bond since the density of the functional groups promoting the adhesion decreases. On the other hand, the adhesion capacity of the hydrogel diminished as the degree of cross-linking increased. In fact, a greater degree of cross-linking restricts segment movement. Consequently, functional groups on polymer chains were unable to migrate to the hydrogel surface to engage with the substrate, hence preventing hydrogel adhesion. Accordingly, alongside the presence of free functional groups that facilitate substrate interaction, the flexibility of the polymer chains constituting hydrogels is essential for hydrogel adhesion [29]. DPH seems to have a minimal impact on the bioadhesive characteristics (Figure 11D). However, a modest enhancement in bioadhesiveness is observed in hydrogels with a higher amount of high molecular weight P(MVE/MA) (H3P(1)), alongside a more significant reduction in bioadhesiveness in the sample containing a greater proportion of thermosensitive polymer (H3P(2)). The electrostatic interaction between the carboxylic groups and DPH induces hydrogel collapse, hence diminishing hydration and the flexibility of the polymer chains, which are essential for bioadhesiveness [30].

### 2.3. Drug Loading and Release Studies

The drug loading efficiency of the hydrogels was assessed for all samples via UV-Vis spectroscopy, exceeding 95% for each sample (95.14% H3p(1), 99.12% H3p(2), 97.13% H3P(1), 96.52% H3P(2)). The high loading efficiency is due to the presence of multipositive charges on the MB molecule that interact electrostatically with the negatively charged carboxylic groups of the hydrogel.

The hydrogels were chosen for their potential use as an implant for drug delivery applications. As follows, a buffer solution at a pH of 7.4 with an increasing amount of triggering agent (DPH) was employed for the tests. Typically, the release rate of drugs can be controlled by the expansion of the polymeric matrix and the movement of the trapped drug through it [31].

**Figure 12:**
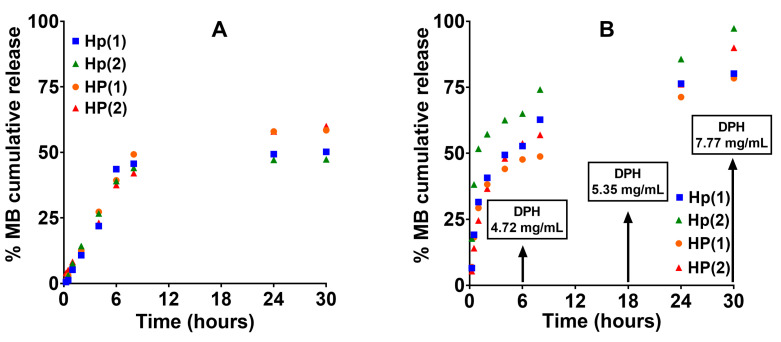
Release profiles of methylene blue from P(NIPAAm-co-HEAAm)/P(MVE/MA) in PBS (**A**) and PBS in the presence of increasing amount of triggering agent (DPH) (**B**) at 37 °C.

As shown in Figure 12, in the first hours, a large amount of drug was released from all tested samples because the polymeric network of the hydrogel, in the presence of small amounts of triggering agent, is still in the swollen state, and the diffusion of the drug is not hindered. Instead, by increasing the amount of triggering agent, the polymer network collapses at the temperature of the human body (see Figure 11B), and the diffusion of the drug is slower.

**Figure 13:**
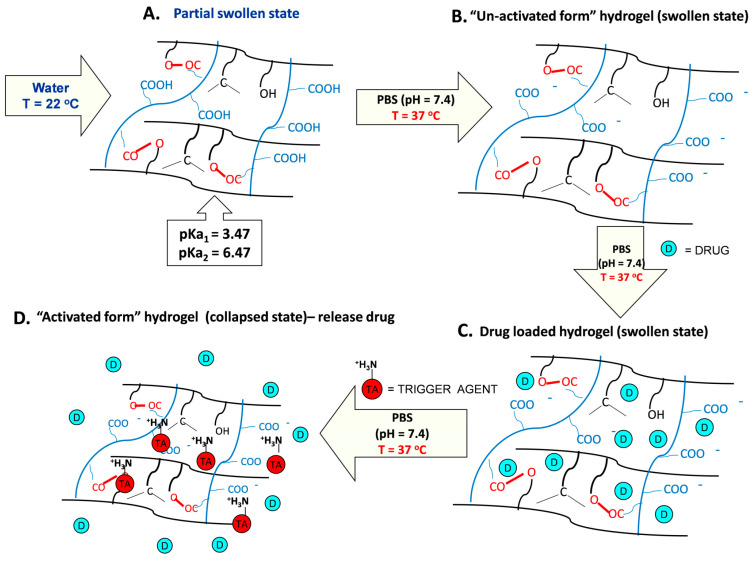
Schematic illustration of the operational principle of HP hydrogel in simulated physiological fluids PBS at pH = 7.4.

It must be underlined that MB has three basic sites, which are represented by the nitrogen atoms in the middle ring and alkyl amine groups. The nitrogen atom in the middle ring is the least basic, as indicated by the pKa values, which are pKa1 = 2.6, pKa2 = 11.2, and pKa3 = 11.2 [32]. On the other hand, DPH has a pKa = 8.98 [12], a lower value than that of DPH, and therefore it is a weaker base. In simulated physiological conditions, PBS at pH = 7.4, both the amino groups of MB and DPH are protonated (ionized) and interact electrostatically with the carboxylic groups of the hydrogel. Even if the amino groups of DPH are less basic, they interact at certain concentrations, with the carboxylic groups displacing the bound MB, setting it free, and activating the thermosensitivity of the hydrogel that will collapse. To enhance comprehension of the operational concept of the smart hydrogel in simulated physiological fluids (PBS, pH = 7.4), a schematic illustration is presented in Figure 13. In water, the hydrogel is partially swollen because the carboxyl groups of the copolymer are protonated, less hydrophilic (Figure 13A). Under simulated physiological conditions (PBS at pH = 7.4), both carboxyl groups (pKa1 = 3.47; pKa2 = 6.47) pass into the ionized, more hydrophilic form, and therefore the hydrogel loses its thermosensitivity (becomes “inert”) (Figure 13B). At this stage, the hydrogel is loaded with the model drug MB (Figure 13C). In the presence of the triggering agent (TA), it interacts electrostatically with the carboxyl groups, and the hydrogel becomes “activated” and collapses, ejecting the incorporated drug (Figure 13D).

## 3. Conclusions

pH/temperature-sensitive hydrogels based on thermosensitive polymer (NIPAAm-co-HEAAm) and pH-sensitive polymer P(MVE/MA) in an appropriate ratio were synthesized through a new solvent-free, thermal cross-linking method. These new hydrogels lose the thermosensitive properties of P(NIPAAm-co-HEAAm) due to the ionization of carboxylic groups (pKa1 = 3.47; pKa2 = 6.47) in simulated physiological fluids at pH = 7.4. However, the hydrogels regain the thermosensitivity after electrostatic interaction of P(MVE/MA) with the triggering agent (DPH) due to modification of hydrophilic/hydrophobic balance. This dual system may serve as the foundation for the next generation of biological active compound delivery systems that can identify biological substances (triggering agents) generated by the organism under pathological situations. Subsequent to electrostatic interactions with the activating agents, the delivery component (thermosensitive hydrogel) undergoes collapse and releases the drugs.

## 4. Materials and Methods

### 4.1. Materials

Poly(methyl vinyl ether-alt-maleic anhydride) (P(MVE/MAn)), with two molecular weights (~210 and ~1000 kDa) from Sigma-Aldrich (Chemie Gmbh, Darmstadt, Germany), was subjected to purification by using an appropriate solvent and subsequently separated by precipitation in methanol to eliminate contaminants. N-isopropylacrylamide (NIPAAm) and hydroxyethyl acrylamide (HEAAm) were purchased from Sigma-Aldrich Chemie Gmbh, Darmstadt, Germany. Potassium persulfate (KPS) and N,N,N′,N′-tetramethylethylenediamine (TEMED) were acquired from Fluka, Buchs, Switzerland. The phosphate buffer solutions (PBS) at pH 7.4 (50 mM NaH_2_PO4 + 40 mM NaOH) were prepared in the laboratory. Methylene blue (MB) and diphenhydramine (DPH) were purchased from Sigma-Aldrich (Chemie Gmbh, Darmstadt, Germany) and were used as model drug and model triggering agent, respectively.

### 4.2. Preparation of Thermosensitive Linear Polymer

P(NIPAAm-co-HEAAm) was synthesized by free radical copolymerization of NIPAAm and HEAAm in water in the presence of potassium persulfate (KPS) and tetramethylethylenediamine (TEMED) as redox initiation systems. Practically, a total of 12 millimoles of NIPAAm and HEAAm in different molar ratios (1:1, 2:1, and 5:1) were dissolved in 10 mL of water. Prior to polymerization, dried nitrogen was bubbled through the solution for 30 min. Then, KPS (1.85% molar ratio versus co-monomers) was added to the mixture, and the solution was purged with dry nitrogen for another 10 min. The reaction mixture was allowed to react at room temperature for 6 h. The polymer solution was dialyzed for 3 days against distilled water at 22 °C (molecular weight cut off 10,000–12,000 Da) and then lyophilized.

### 4.3. Preparation of Thermally, Solvent-Free Cross-Linked Hydrogels

The aqueous solutions containing different weight ratios between P(NIPAAm-co-HEAAm) and P(MVE/MAn) have been obtained at a final concentration of 3% *w*/*v*. Two different molecular weights of P(MVE/MAn) have been chosen for the cross-linking study, low (210 kDa) and high (1000 kDa), marked with a small and capital p, respectively, in the sample codes. In Table 2 is shown the composition of the initial mixture. Subsequently, the solutions were poured into the 24-well plates, placed in a refrigerator, and freeze-dried for 2 days. The resulting samples were obtained in cylinder-like pieces. These samples are not cross-linked, and therefore they are soluble in water.

In order to obtain cross-linked hydrogels, the samples were subsequently placed inside the oven for 8 h at 120 °C. To examine the effect of temperature protocol on cross-linking, some hydrogel samples were subjected to two temperature cycles, each lasting 8 h. The resulting hydrogels were washed in a large amount of water to remove the soluble fraction of polymer residues. The hydrolysis of the anhydride bonds occurs during washing, resulting in the stabilization of the hydrogel network by only the ester cross-links.

### 4.4. Determination of the Copolymer Composition

^1^H-NMR spectra of P(NIPAAm-co-HEAAm) and polymer mixtures were obtained in deuterated water using a Bruker Avance NEO 400 Spectrometer (Bruker BioSpin, Ettlingen, Germany) operating at 400.1 MHz for 1H, with a 5 mm four nuclei direct detection z-gradient probe using standard pulse sequences, as delivered by Bruker with TopSpin 4.1.3 spectrometer control and processing software. Chemical shifts are reported in δ units (ppm) and were referenced to the sodium 3-(trimethylsilyl)-[2,2,3,3-d4]-1-propionate (TSP) internal standard at 0.0 ppm. NMR spectra were recorded at temperatures between 25 and 50 °C.

### 4.5. Determination of the Lower Critical Solution Temperature (LCST)

The LCST was calculated based on the correlation between the change in absorbance at 450 nm and temperature. The UV–Vis Specord 200 spectrophotometer supplied with a temperature controller (Analytic Jena in Jena, Germany) was utilized. A 1% (*w*/*v*) polymer solution (each separately or in a mixture) was obtained in a standard phosphate buffer solution (PBS) with a pH of 7.4 (50 mM Na_2_HPO_4_ and 40 mM NaOH). The heating rate was set at a constant value of 0.2 degree Celsius every 3 min. The LCST is defined as the temperature at which the absorbance reaches a value of 0.5 [12].

### 4.6. Characterization

#### 4.6.1. Fourier Transform Infrared Spectroscopy (FTIR)

The chemical structure of the copolymers and hydrogels was analyzed using attenuated total reflectance Fourier Transform Infrared Spectroscopy (ATR-FTIR) with an FT-IR Vertex 70 spectrophotometer (Bruker, Wien, Austria). The samples were examined in their solid (lyophilized) form on a KRS-5 substrate using a frequency range of 4000–400 cm^−1^. The data processing was conducted with the OPUS 6.5 software, developed by Bruker Optics.

#### 4.6.2. Conductometric Titration

The carboxyl groups present in hydrogels were accurately measured using conductometric titration. A Radiometer CMD 210 conductivity meter (Radiometer, Copenhagen, Denmark), equipped with a CDC 865 cell, was utilized. Previously, the hydrogels were weighed and then immersed in an excess amount of 0.1 M NaOH aqueous solution. Once the ion exchange process reached a state of balance, the samples were extensively washed with distilled water, and the collected solution was titrated with 0.1 M HCl. Exchange capacity (E.C.) (meq COOH/g hydrogel) was calculated according to Equation (2):

(2) $$E.C.=\left[\left(V_{NaOH}-V_{HCl}\right)\times 0.1\right]/a,$$

where *V*_NaOH_ represents the volume of 0.1 N NaOH, *V*_HCl_ represents the volume of 0.1 N HCl, and *a* represents the mass of the sample (g).

#### 4.6.3. Cross-Linking Degree (C.D.)

Cross-linking degree of the hydrogels was assessed based on determination of the carboxylic groups (exchange capacity) before and after the cross-linking procedure. The exchange capacity was determined by the same procedure as explained before [22]. C.D. (%) was calculated according to Equation (3):

(3) $$C.D.\;(\%)=\left[\left({E.C.}_{i}-{E.C.}_{f}\right)/{E.C.}_{i}\right]\times 100,$$

where *E.C._i_* and *E.C._f_* represent the exchange capacity of the hydrogel before and after the cross-linking procedure, respectively.

#### 4.6.4. Swelling Kinetics and Volume Phase Transition Temperature (VPTT) Determination

The dry hydrogel samples were weighed and subsequently immersed in an excess of PBS solution at 37 °C. The hydrogel was subsequently extracted from the medium at specified time intervals and weighed following the blotting of excess surface solution with moistened filter paper [27]. The swelling ratio (SR) was determined using Equation (4):

(4) $$SR=\frac{W_{s}-W_{d}}{W_{d}},$$

where $W_{s}$ is the weight of swollen hydrogels at each time and $W_{d}$ is the weight of dry sample.

In order to establish the VPTT of the hydrogels, the swelling ratio (SR) was determined over 25–50 °C range with a heating rate of 3–5 °C increments using a thermostatted water bath. The dry samples were initially weighed and subsequently immersed in buffer solution to equilibrate for 12 h at each specified temperature. Prior to each weighing, the samples were wiped with filter paper to eliminate the excess solution.

The VPTT of the hydrogels was identified as the inflection point in the swelling ratio-temperature curve through Boltzmann fitting of the experimental data [12].

#### 4.6.5. Bioadhesiveness Tests

The mucoadhesive property was assessed using a Brookfield Texture PRO CT3(R) texture analyzer (Brookfield Engineering Laboratories Inc., Middleboro, MA, USA). The experiment utilized a chicken skin mucosa as the surface model affixed to the underside of the probe, which has a diameter of 10 mm, using double-sided adhesive tape. The hydrogel, which was swollen in physiological simulated fluid (phosphate buffer solution at pH 7.4, PBS), was also attached to the holder using double-sided adhesive tape. The experiment was initiated by lowering the probe onto the hydrogel at a predetermined pretest speed. Once the trigger force was reached, the probe containing the hydrogel was applied to the mucosa at a speed of 0.5 mm/s. It was then maintained in place for 120 s with a force of 1 N. The maximum force (Fmax) needed to detach the hydrogels was determined at the same speed of 0.5 mm/s.

### 4.7. Drug Loading/Release Experiments

Methylene blue (MB) was used as a model drug in loading and release investigations on/from hydrogels. For loading, approximately 50 mg of the dried sample of hydrogel was immersed in a 5 mL aqueous solution containing 0.1 mg/mL of MB. The sample was then left at room temperature for 3 days. Subsequently, the samples containing the drug (HP-MB) were extracted, cleansed with distilled water, and desiccated at room temperature under vacuum in an oven. The remaining amount of MB in the collected aqueous solutions was measured using UV spectrophotometry utilizing a calibration curve that had been prepared beforehand. The drug loading efficiency (DLE) is calculated by subtracting the final quantity from the initial quantity of MB in the solution where the hydrogels were immersed.

The amount of MB at equilibrium in each sample (q_e_) was determined according to Equation (5):

(5) $$q_{e}=\left[\left(C_{0}-C_{e}\right)\times \left(V/m\right)\right]\times 10^{3}$$

where *C*_0_ is the initial concentration of the drug solution (mg/mL), *C*_e_ is the concentration of the drug at equilibrium (mg/mL), *V* is the volume of the drug solution (mL), and *m* is the amount of the hydrogel sample (mg).

MB release studies were performed by immersing loaded HP-MB samples (~50 mg) in 25 mL phosphate buffer solutions at pH 7.4 and 37 °C in the absence/presence of diphenhydramine (DPH), taken as a thermosensitivity-activating (triggering) agent. At specified time intervals, a volume of 3.0 mL of the release buffer was extracted and the concentration of the drug was measured using UV-Vis spectrophotometry using a calibration curve that was previously prepared. An equal amount of fresh release buffer containing 10 mg/mL DPH was added to the drug-buffer solution in order to maintain the volume of the release fluid unchanged and to trigger the collapsing of the hydrogel.

## Figures and Tables

**Table 1:** Feed and copolymer composition and the influence of the co-monomer molar ratio on the LCST (concentration of the copolymer solution was 1%, *w*/*v*).

**Sample** **Code**	**Co-Monomer Composition**				**LCST ^2^** **(PBS at pH 7.4)**
	**In the Feed ^1^** **(% Molar Ratio)**		**In Copolymer** **(% Molar Ratio)**		
	**NIPAAm**	**HEAAm**	**NIPAAm**	**HEAAm**	
H1	50	50	50.77	49.23	71.1 ± 0.21
H2	66.7	33.3	65.79	34.21	49.08 ± 0.25
H3	83.3	16.7	81.97	18.03	38.35 ± 0.18

^1^ The total moles of the co-monomers in the feed were maintained at 12 × 10^−3^ moles for all samples. ^2^ Data are the results of three independent experiments.

## Data Availability

The original contributions presented in this study are included in the article. Further inquiries can be directed to the corresponding authors.
